# Gene expression in the Parkinson's disease brain

**DOI:** 10.1016/j.brainresbull.2011.11.016

**Published:** 2012-07-01

**Authors:** Patrick A. Lewis, Mark R. Cookson

**Affiliations:** a Department of Molecular Neuroscience, UCL Institute of Neurology, Queen Square, London WC1N 3BG, United Kingdom; b Laboratory of Neurogenetics, National Institute on Aging, Bethesda, MD 20892, USA

**Keywords:** Parkinson's disease, Microarray, Gene expression, Neuropathology, Genome wide

## Abstract

The study of gene expression has undergone a transformation in the past decade as the benefits of the sequencing of the human genome have made themselves felt. Increasingly, genome wide approaches are being applied to the analysis of gene expression in human disease as a route to understanding the underlying pathogenic mechanisms. In this review, we will summarise current state of gene expression studies of the brain in Parkinson's disease, and examine how these techniques can be used to gain an insight into aetiology of this devastating disorder.

## Highlights

► Summary of the existing literature on gene expression in Parkinson's disease, concentrating on alterations in gene expression in the brain. ► Analysis of the strengths and weaknesses of a genome wide approach to assessing gene expression in Parkinson's. ► A preview of what lies ahead for gene expression in Parkinson's disease as technology advances.

## 1 Background

Parkinson's disease (PD) is a neurodegenerative movement disorder characterised by resting tremor, bradykinesia, stiffness of movement and postural instability. These movement problems are largely a consequence of substantial cell loss in the *Substantia nigra pars compacta* and concomitant loss of dopamine (DA) neurotransmitter, and of the deposition of protein within the brain as intracellular inclusions called Lewy bodies [68].

Over the past fifteen years, an increasingly detailed knowledge of the genetic factors that contribute to PD has emerged [49]. There are now a number of identified *PARK* loci that are linked to autosomal dominant or recessive forms of disease. Furthermore, in the last 24 months several genome wide association studies have opened the door to a wider understanding of genes involved in the sporadic form of the disease [46,49,96,98,104,110]. Despite these advances, the mechanisms underlying cellular degeneration in the brains of people with PD remain poorly understood.

The development of genome wide approaches for analysing gene expression may yield insights into the underlying mechanisms causing PD. By examining the expression of genes within the brains of people with disease *post mortem*, and comparing these data to controls without PD, it is hoped that expression signatures for disease can be identified [90]. This, in turn, could highlight pathways that lead to dysfunction – an approach that has yielded important advances in other disorders [128]. In this review, the importance of gene expression in PD will be examined using the α-synuclein gene and MPTP toxicity as pathfinders for the more complex analyses facilitated by whole genome analysis. The current status of such studies using brain samples from patients will then be summarised and discussed with a view to the future directions that may be helpful to expand this work.

## 2 *SNCA* and *MAPT* expression in Parkinson's disease

That gene expression is a crucial component of the disease process in Parkinson's can be illustrated using the example of α-synuclein. This gene was first linked to PD with the identification of an A53T mutation in *SNCA* (the gene that encodes the α-synuclein protein) as the causative insult in a large family of Mediterranean origin with autosomal dominant inherited Parkinson's disease [92]. Soon after this, α-synuclein was identified as a major component of Lewy bodies, suggesting that the same protein is also linked to sporadic PD [111].

A key discovery that supports the role of wild type α-synuclein in inherited and sporadic PD was the identification of a triplication of the *SNCA* locus, resulting in a doubling of α-synuclein expression, in a family with inherited parkinsonism and dementia [78,107]. Subsequently, several additional families were reported where duplications or triplications of the α-synuclein gene were found [16]. Intriguingly, there appeared to be a correlation between the gene dosage and the severity of disease [39,57]. These studies provided substantive evidence that large increases in the expression of α-synuclein can result in neurodegeneration, and that the amount of expression is negatively correlated with age at onset and positively correlated with disease severity.

Further evidence that there may be a connection between the level of α-synuclein expression and risk for development of PD outside of rare families has recently been provided by genome wide association studies. Common genetic variation at the *SNCA* locus has been identified as one of the most consistent genetic risk factors for PD [46,98,104,110]. This suggests a model where the level of α-synuclein expression level in an individual's brain is a significant determinant of risk for neuronal degeneration.

Another important gene for PD is *MAPT*, which encodes the microtubule associated protein tau. Deposition of Tau is seen in Alzheimer's disease as tangles, but is not generally seen in PD [129]. Despite this, the *MAPT* locus has been identified as a major risk factor for PD in several genome wide association studies. Using data from human brain, it has been shown that the risk alleles for PD are associated with higher levels of expression of *MAPT* than protective alleles [104]. Therefore, like *SNCA*, too much *MAPT* increases the risk for PD.

*SNCA* and *MAPT* are important examples of the link between the expression of individual genes and PD that has been elucidated by the interaction of clinical medicine, genetics and neuropathology. However, it remains likely that there will be additional influences on pathogenesis that might be rather subtle and may not be measurable at the level of DNA. Using genome wide expression analysis approaches may allow a broader and much more rapid approach to identifying additional genes, families of genes and pathways that are linked to neurodegeneration in Parkinson's. In the following sections we will identify several linked questions related to the pathophysiology of PD and discuss where gene expression profiling may be able to add some novel insights.

## 3 Gene expression, regional development and vulnerability

Gene expression may be a clue to the solution of one of the major unanswered questions in the study of PD, and indeed in neurodegeneration as a whole: the regional preferential vulnerability exhibited by neurons within the brain. In PD, a substantial proportion of neuronal death, but not all, is localised to dopaminergic neurons in the *Substantia nigra* [11,27,35]. Surveys of the pathology in PD suggest that there are distinct patterns in the spread of cell loss and Lewy body burden [12,27].

The reasons for this pattern of degeneration are unknown but can be hypothesised that underlying variability in gene expression across the brain that specifies cellular phenotypes and roles is important [13,77]. Precisely how gene expression, regional and cellular identity map on to patterns of neurodegeneration is a matter of debate [28,102,114]. One example of a link between specific gene expression and neurodegeneration is given by neurotoxicity linked to 1-methyl-4-phenyl-1,2,3,6-tetrahydropyridine (MPTP). This chemical first came to light in reports of intravenous drug users exposed to MPTP who developed a parkinsonian syndrome [66,67]. Subsequent investigations demonstrated that the reason for this toxicity was due to conversion of MPTP to a toxic cation, 1-methyl-4-phenylpyridinium (MPP^+^) by monoamineoxidase B in glial cells within the brain, and the selective uptake of this toxin by the dopamine transporter by doperminergic neurons [18,19,59,120]. This is a clear example of how a specific combination of gene expression in the presence of an acute environmental insult can lead to a definite pattern of neurodegeneration. The principle of regional gene expression and patterns of neurodegeneration, however, is one that can be extended and explored in much greater detail using genome wide expression studies.

## 4 General aspects of gene expression analysis in the PD brain

Technologies to examine whole genome gene expression have advanced rapidly in the 15 years since the first application of microarray technology to the analysis of gene expression [71]. The techniques now available include microarray based gene-by-gene expression, exon microarray analysis and transcriptome RNA sequencing [4,5,20,60,71,81]. All of these techniques offer genome wide coverage, with increasing depth of information revealed moving from simple arrays to exon arrays to sequencing. A detailed review of the technology underlying these approaches is available elsewhere in this special issue. The literature describing brain gene expression studies in the field of Parkinson's disease and related disorders is summarised in Table 1. Individual studies will be discussed below, but it is first important to consider some of the general aspects of gene expression studies in the context of a neurodegenerative disease.

There are a series of general strengths and the weaknesses of the genome wide expression approach. The main strength is an unprecedented power to look at gene expression across the genome in a relatively unbiased manner using small amounts of material. One limitation, highlighted by many authors and common to most gene expression studies, is that is difficult to separate out cause and effect. That is, do the changes in gene expression indicate an underlying cause of the disease, or do they represent a downstream consequence of the underlying aetiology. While both are of interest it is important to be able to discriminate between them [91].

Another limitation is that studies such as this are dealing with a mixed cell population, and a population where one set of samples has suffered massive cell loss. Because of this, we need to consider which brain region(s) we should examine. Specifically, should expression in the *Substantia nigra* as a whole be assessed by extraction of mRNA from whole brain tissue, or should investigations be focused on specific neuronal types. The advent of single cell expression analysis, facilitated by the development of laser capture microdissection (LCM), has been especially important for the latter [9,32,95]. Some of the studies discussed below have therefore focussed on the surviving dopaminergic nigral cells left at the endpoint of disease in the brain [11].

One alternative approach is to examine gene expression outside of the *Substantia nigra*. This merits consideration because although the amount of neuronal cell loss is less dramatic in other area, there is still Lewy body pathology in a range of brain regions. If we consider PD to be a multi-brain region disease, then there might be important insights from considering where gene expression differences occur weighting for the contribution to pathology.

A further factor to be taken into consideration when examining global gene expression in Parkinson's is the underlying clinical heterogeneity of the disease, and the emerging realisation that the boundaries between PD and other neurodegenerative disorders are not as distinct as was once thought [94]. Within the diagnosis of PD itself, it is increasingly recognised that a substantial proportion of cases present with a dementia component in addition to the classical movement disorder phenotype (Parkinson's disease dementia or PDD) [1]. Dementia with Lewy bodies (DLB) is an overlapping disorder where the presenting phenotype is dementia rather than a movement disorder although by the later stages of disease the distinctions between PD, DLB and PDD are blurred [70,76,86]. In addition, there are the related disorders multiple system atrophy (MSA) and progressive supranuclear palsy (PSP), which have substantial clinical overlap with PD but are distinct clinical and pathological entities [109]. Mutations in genes such as *MAPT* also have parkinsonism as part of their clinical presentation [30,56,117].

This is relevant for gene expression studies as a careful appraisal of clinical and pathological details is required for brain tissue to be included in studies of PD. The inclusion of disorders with divergent phenotypes and differing pathologies could increase the complexity of data sets that, by their very nature, have a low signal to noise ratio [127]. The heterogeneity and overlap between neurological disorders should, however, be recognised as an opportunity as well as a confounding factor, as examining the commonalities between gene expression alterations in these disorders is potentially illuminating with regard to the disease process in individual disorders [42].

Further to this, a major confounding factor is the underlying variation in gene expression due to the different genetic backgrounds of the samples [113]. Many of the studies performed to data have matched cases and controls for known methodological and biological variables (age, gender and *post mortem* interval are the most commonly considered), but this does not address the potential for undetected bias in genetic background to introduce either false positive or false negative results [21,31,63].

Studies using brain tissue from neurologically normal controls have shown there are many common genetic variants in the human genome that show a statistical association with expression levels, usually of nearby genes [41]. Perhaps surprisingly, the number of transcripts where there is an effect of genotype on expression outweighs the number where age or gender shows an effect. Relatively few studies have attempted to match for genetic variation and it would be difficult to do so without having very high numbers of samples. However, it is helpful to bear in mind that even the most carefully matched sample series are usually quite modest in number and are not matched for all known variables.

Following from this, it is important to note which genes can be replicated across studies. Genes whose expression differ between PD and controls are often highlighted when they are replicated across studies but in practise many more genes are not replicated, leading to a bias in reporting that hinders critical review. One problem with attempting to perform meta-analysis of array studies in particular is that probe designs vary across platforms and are often not stable even for multiple iterations of the same array product. Therefore, in the following section we will note where consistency has been found but will limit comments on lack of replication to specific instances where non-array techniques have been used.

In the following sections, we will discuss some of the major approaches that have been taken in examining PD gene expression, including homogenised tissue compared to laser captured individual cells and to material outside the brain.

## 5 Gene expression in bulk extracts of the *Substantia nigra*

The first description of genome wide expression analysis of PD brain tissue was published in 2004 by Grunblatt and co-workers [43]. A case:control approach was taken to look for differences in gene expression between brains from seven neurologically normal individuals and seven PD cases. Whole RNA was extracted from nigral tissue and gene expression was assessed by whole genome affymetrix expression arrays.

Analysis of the data from these extracts revealed a large number of genes up or down regulated between cases and controls (137 in total), using a threshold of >1.5-fold alteration and deeming a p value of <0.05 to be significant. To decrease the number of genes under consideration, the authors carried out a second sifting of the results by limiting the genes studied to those that showed at least a 1.5-fold alteration in 5 out of 6 parkinsonian samples. This limited the genes to 20, 3 of which were genes down regulated in the PD brain linked to DA transmission and metabolism, a finding that was perhaps to be expected given the widespread DA neuronal loss in these samples (see above). The same study uncovered a number of down regulated genes linked to protein degradation, including the E3 ligase SKP1A. This is of particular interest given the fact that several genes linked to genetic forms of Parkinson's disease are associated with protein degradation [49].

Hauser and co-workers took a different approach, taking advantage of the fact that there are multiple heterogeneous disorders linked to parkinsonism as a tool to differentiate disease specific gene alterations [53]. By extracting mRNA from brain tissue isolated from patients with PD, PSP and FTDP, along with control samples, the authors hoped to dissect expression differences specific to each disease. The authors were able to identify genes (12 in total) that displayed altered expression across all three disorders compared to controls. Based upon the hypothesis that these were more likely to be linked to generalised neuronal degeneration as a phenotype secondary to the underlying aetiology, exclusion of these common genes allowed the hierarchical segregation of the disease groups – a statistical separation that was not possible if these genes were included. Several cellular pathways were identified as being altered in the PD samples including those linked to protein misfolding and degradation, and nuclear encoded mitochondrial genes.

Noureddine and colleagues combined loci identified by a genome wide association study that they had previously published with gene expression data harvested from 3 PD brains and 2 control samples [69,85,101]. This study focused on a number of genomic regions identified in their genome wide association studies as being linked to age at disease onset and absolute risk for disease, an approach the authors dub serial analysis of gene expression, following up on an earlier publication examining gene expression in control *Substantia nigra* [52]. This is a potentially very powerful method to dissect the genetic and expression pathways that lead to neurodegeneration in PD. However both the association study and the expression study in this case suffer from low sample number. It is only recently, with the advent of association studies with case control numbers in the thousands, that replicated risk loci have been identified [40]. Using analysis of ESTs, the authors identify a number of loci within the regions previously identified by their association studies, and note an up regulation of mitochondrial encoded genes compared to nuclear encoded mitochondrial genes in the PD brains compared to controls.

The studies discussed above have examined the expression of mRNA but this only represent a portion of the transcriptome. There are a small number of studies examining small RNAs in the PD brain. Kim et al. compared miRNA expression in the *Substantia nigra* of PD cases and controls and identified mir-133b as being differentially expressed between cases and controls [62]. They further suggested that mir-133b regulates Pitx3, a transcription factor involved in differentiation of mature dopaminergic neurons, thus indicating that mir-133b may have cell-specific roles. More recently, Minones-Moyano and co-workers carried out a study of 11 PD brains compared to 6 pooled control brains looking specifically for miRNAs associated with the disease state, uncovering miR-34b/c as downregulated in PD samples [79]. The potential role for miRNAs in PD has recently been reviewed, pulling together research from patient samples and the more extensive literature from model systems [50].

## 6 Examining brain regions other than the nigra

Several other studies have compared gene expression in PD tissue to tissue from the brains of people afflicted by related, but separate, disorders. Vogt et al. compared MSA to PD and control tissue, with the aim of identifying genes differentially expressed specifically in MSA [124]. They found that GPR86 and RGS14, both associated with G protein signalling, had lower expression in both MSA and PD putamen. In contrast, Langerveld et al. directly compared gene expression in the rostral pons between 7 MSA brains and 5 control brains [65]. Interestingly, this study identified changes associated with MSA in a number of pathways that have also been implicated in alterations within the *Substantia nigra* in PD – notably down regulated genes linked to the mitochondria and the ubiquitin/proteasome system – despite the different anatomical origin of the tissue and the different pathology associated with these two disorders. A number of up regulated genes linked to oligodendrocyte maintenance were also identified, which is potentially significant as one of the major pathological features of MSA that is distinct from PD is the presence of α-synuclein pathology in oligodendroglia.

Finally, Grunblatt and colleagues stepped outside Lewy body disorders altogether to compare brain gene expression between PD and AD [44]. The study was primarily focused on AD, and therefore examined tissue from the hippocampus, gyrus–frontalis–medius and the cerebellum, regions differentially impacted in this disease [26]. They identified 12 genes that were altered in AD and PD tissue, along with 4 that displayed differential expression between AD and PD (and control) – suggesting disease specificity. Intriguingly, one of these genes was BACE1, which is involved in the proteolytic processing of the Amyloid Precursor Protein to produce the Aβ peptide, which was down regulated in AD and upregulated in PD [123].

Two studies from 2005 and 2006 approached the question of regional vulnerability in PD by examining differential gene expression from multiple brain regions. Zhang and colleagues investigated three brain regions in 15 PD and 15 control brains [131]. They identified a number of transcripts that were altered across the *Substantia nigra*, putamen and Brodmann area 9, verifying the top fold-changed genes by rtPCR and *in situ* hybridisation. These three regions were specifically chosen as being primarily affected, secondarily affected and not involved in PD. Statistical and pathway interrogation of the results highlighted electron transport chain and ubiquitin/proteasome system genes as being differentially expressed in the tissue most impacted in Parkinson's disease.

Papapetropolis et al. spread the net a little wider, and studied a total of 21 different brain regions in 22 PD and 23 control brains [89]. This group had a slightly different approach, and were specifically looking for genes that were differentially regulated across the regions examined. However, no genes were altered in the same way across all 21 brain regions and so the authors lowered the stringency of their search, discovering 11 genes that were regulated in a similar manner across 18 of 21 brain regions in cases versus control. They focused on one of these, the mitochondrial ribosomal protein S6, *MRPS6*, a nuclear encoded gene that was found to be expressed at higher levels in PD brains compared to controls.

## 7 Isolating neurons using Laser capture microdissection

Most of the above studies used bulk extractions from tissue to generate RNA for microarray analyses. As discussed above, a difficulty with this approach is that the cellular composition of a brain where neurodegeneration has been active differs from disease-free controls. It may therefore be very helpful to examine specific cell types to more directly compare controls and cases and there are techniques such as Laser capture microdissection (LCM) that allow for small numbers of cells to be isolated from tissue that are suitable for array analysis. Additionally, there is a great deal of information that may be etiologically relevant to the disease process that can be seen in surviving cells. Lewy bodies, an important clue for pathogenesis, were first identified by histological, and later by immunological, techniques examining pathology at the cellular level. Because of these reasons, examining gene expression in isolated neurons has been attempted by several groups.

Lu et al. used a combination of LCM and PCR fingerprinting to examine cells from five PD brains comparing cells containing Lewy bodies to those without [72]. The role of the Lewy body is PD is controversial in that it is not clear if the formation of pathological structures is a toxic event or an attempt by the neuron to respond to damage and thus represents protection. Equally Lewy bodies could be both toxic and protective in different contexts or might be neutral. The analysis of expression by Lu et al. used PCR analysis of expressed sequence tags (ESTs) to reveal that neurons without Lewy bodies had significantly higher expression of genes linked to cell survival compared to Lewy body containing neurons. This suggests that these cells were in better health than their aggregate containing companions and argues against a model where Lewy bodies act as a protective cellular response to an insult from aggregated α-synuclein [22,116].

Similar to these studies, a number of groups have examined the subtleties of contrasting gene expression in Parkinson's disease dementia (PDD) and PD. Stamper et al. [112] compared gene expression in cortical neurons isolated by LCM from 14 control brains, 13 PDD and 15 cognitively normal PD brains, identifying significant differences in gene expression between the two disease series. The key pathways they identified were axonal transport, cell adhesion and mRNA splicing.

The authors also demonstrated how the same data set could be used to asked different experimental questions by comparing the PDD and PD samples. Based on the hypothesis that PDD represents a natural continuation of PD, and that cognitively normal PD brains will contain the early markers of degeneration within the cortex that would have resulted in degeneration and dementia had the patient survived the disease process for a longer period [2,61].

A very different way to examine regional and cellular vulnerability was taken by Lu and colleagues, who compared gene expression profiles of dopaminergic neurons captured by LCM from the *Substantia nigra* (which are devastated in PD) and the central grey area (which is almost completely spared) in control brain samples [73]. Little evidence of differential gene expression was discovered between the two populations of DA neurons. The authors also refer to the fact that, despite similar gene expression profiles, the neurons from these two areas have differing electrophysiological properties and connectivity within the brain.

Moran and co-workers carried out a set of experiments based on the same concept using whole tissue rather than laser-captured neurons [82,134,135]. This study took advantage of the severe damage caused to the lateral *Substantia nigra* in PD compared to the relative sparing of the medial part of this region [35]. This study identified a large number of genes altered throughout the *Substantia nigra*, with a number that were specific to either the lateral or the medial region. One key finding was the identification of homocysteine-inducible, ER stress inducible, ubiquitin like domain member 1 (HERPUD1) as being up regulated in PD tissue compared to control. Several other alternatively expressed genes also linked in to protein folding and degradation pathways, again highlighting the potential relevance of these pathways to PD.

Several groups have used genome wide expression data from Parkinson's patient brains to examine the role of gender in the disease process. Epidemiological studies have shown that gender is a significant risk factor for PD, with men having a two fold higher incidence than women [6,121]. Both Cantuti-Castelvetri et al. and Simunovic et al. examined alterations in gene expression stratified by gender in neurons from the *Substantia nigra* [15,106]. Both studies report gender dependant alterations in gene expression within the *Substantia nigra*, both in PD and control brain. The former study highlighted kinase signalling pathways, proteolysis and WNT pathways as being altered between males and females, as well as upregulation of PINK1 and α-synuclein in males compared to females. The latter study, following up on previously published research from the same group, highlighted mitochondrial function genes as being upregulated in the male brain, suggesting a potential route to increased incidence in males, although only a small number of samples were used for each group [105]. Interestingly, the Simunovic study attempted to re-analyse the data from the Cantuti-Castelvetri paper using different statistical analysis techniques to those originally used and were unable to generate a dataset that could be used, emphasising the importance of statistical processing in the interrogation of gene expression data [29,83,132].

More recently, Zheng and collaborators – a large consortium of groups carrying out gene expression studies of PD – carried out a meta-analysis of existing data on gene expression in PD, along with a number of new data sets [133]. By combining data from tissue and LCM isolated neurons, they were able to include a total of 172 unique PD samples and 139 unique control samples. An interesting subset of the data examined in this study consisted of expression data from the brains of 16 neurologically normal individuals with incidental Lewy body disease, possibly representing a preclinical stage of disease [3,38]. Carrying out pathway analysis, the authors were able to identify 10 gene sets that were consistently and significantly altered between cases and controls. These included genes involved in the electron transport chain, mitochondrial biogenesis, glucose utilisation and glucose sensing.

Importantly, genes under the control of the master regulator PGC-1 α were under expressed in Parkinson's tissue and neurons isolated from Parkinson's *Substantia nigra* compared to controls. To test the impact of PGC-1 α a series of experiments were carried out in primary rat neurons in culture, examining the impact of modulating this gene in two experimental models for PD:α-synuclein and rotenone toxicity. In both systems, PGC-1α was able to suppress toxicity, providing experimental evidence to support the role of this gene in the disease process in PD. In a fascinating recent report, PGC-1α has been linked to the process of aging and telomeric shortening – perhaps providing a unifying theme for the process of neurodegeneration [97].

## 8 Gene expression outside of the brain

The brain is not the only tissue type where the association between disease state and gene expression has been examined. For example, performing expression analysis in blood has been proposed as a way to develop novel biomarkers for PD [45,99,103]. Of interest, Shehadeh et al. have suggested that *SRRM2*, a component of the spliceosome, is differentially spliced comparing PD and control cases in both brain and blood. This study used exon arrays, a technique whereby individual exons are interrogated that can give a semi-quantitative view of alternate splicing in different samples. Although there are no published results using exon arrays in the PD brain, this result suggests that looking at splicing in PD versus controls might be an important line of research in the future, which will be discussed below.

## 9 The benefits and drawbacks of genomic expression analyses in PD

Whole genome approaches have many benefits, particularly the equal weighting given to large numbers of genes. As noted above, data can be gathered from multiple brain regions, cell types and disease pathologies to explore the differences and commonalities between these samples, expanding our understanding of the underlying aetiology of PD.

There are, however, a number of problems when carrying out or interpreting these studies. One major issue is that of sample number, a problem that genome wide association studies have battled with for a number of years [14]. The fundamental problem is that there is limited power to detect relatively small differences in the context of the need to correct for large numbers of multiple tests performed. The ultimate resolution of this issue is to increase numbers of cases and controls, balanced against the cost of this effort. For example, successive genome wide association studies using increasing numbers of samples have revealed greater and greater detail of the genetic architecture of this disease [40,48]. For mRNA measurements, these issues are perhaps more acute as DNA is largely invariant but mRNA is much more dynamic in expression levels. These variables make power calculations in expression studies a fraught topic, even more so than for association studies [118]. Even with the limited number of samples analysed across laboratories, meta-analyses can be performed to improve robustness of results. However, as the raw material for brain gene expression studies is available only *post mortem* there are a large number of quality control problems that are important to attend to [36,58,126].

Another approach to increasing the signal in such studies is to focus on genetically defined PD cases. This has become feasible due to the discovery of increasing numbers of genetic cases in brain banks and in the patient population. For example, several studies have recently examined the impact of mutations in LRRK2 on gene expression in the brain [10,24]. Although potentially informative with regard to the biology of the specific gene under consideration, it is less clear whether such studies will be helpful for understanding alterations in the pathogenesis of sporadic disease.

A central problem specific to the disease tissue samples, and one that is difficult to control for is that there are differences in cellular composition between cases and controls. Since the neuronal loss in the disease impacted areas is extensive, with the majority of cells having died by the time the patient reaches the end stages of disease, there are very few remaining cells in the tissue for mRNA to be extracted from [35,87]. Those cells that remain may have an expression profile that reflects a dying cell, rather than the underlying insults that led to disease originally. In most neurodegenerative conditions there is some reactive astrogliosis and microglial activation. Sampling subsets of cells using LCM overcomes many of these difficulties, but it remains ambiguous whether the cells remaining in an advanced neurodegenerative disease brain are resistant to the disease process due to their phenotype or altered survival pathway expression. Staging of brains may be helpful in identifying early pathological events, but even then the amount of pathology in early stage disease may still be extensive. There are not many practical solutions to these problems if we wish to study the human disease although meta-analytic approaches would appear to be a very promising strategy [133].

An added set of confounding factors that apply as much to control tissue as to disease samples, are the variations in brain state at the point of death and the nature of *post mortem* handling. One widely used measure of tissue quality is *post mortem* interval, the time between death and freezing of brain tissue. The samples described in papers referred to throughout this review possess widely varying *post mortem*. To cite one example, the study by Lu et al. in 2006 had *post mortem* delays of between 7 and 47 h [73]. Attempts have been made to decrease the length of delay for brain bank samples, for example by the Sun Health Research Institute Brain Bank in Phoenix, Arizona [8]. While such efforts are to be lauded, there is not a simple relationship between length of *post mortem* delay and the quality of RNA isolated from brain samples [17,33,51,93]. Whether *post mortem* delay has an impact on the pattern of gene expression is less clear with some analyses suggesting that it may have a measurable effect [7,37].

Factors that are better predictors of RNA quality are the agonal state of the individual, particularly if a person died in a manner where the brain was relatively anoxic for a period of time. A surrogate measure of agonal state is the pH of the brain *post mortem* [7,51,80,93], although this is not always used. The majority of the studies described in this review use one of a number of quality control measures to account for degradation of RNA, for example assessing RNA integrity number (RIN) developed by Agilent technology [100]. How well this correlates with the quality of RNA quality across the transcriptome is not well studied, although a recent study suggests that there might not be a direct relationship between the two [119]. In addition, a remaining concern is that cause of death in neurologically normal individuals tends to be different (heart attacks and cancer are common) to PD patients, and so agonal state and assignment into cases and controls can be correlated, confounding analysis [55].

All of these factors combine to make the analysis of gene expression in the PD brain a particularly challenging task. However, appropriately powered sample series can be constructed with moderate power where age, gender and biological measures of brain mRNA integrity can be reasonably controlled for. Surprisingly, in a large series of control brains where we have measured expression, many of the above parameters (*post mortem* delay, age and gender) have smaller effects on gene expression than some technical variables. There are clear differences in expression between samples from the same brain region submitted from different brain banks. However, the largest effect, even after robust normalisation approaches is the effect of batch of hybridisation of the arrays. This effect is not well studied, and is only apparent in larger series. One pragmatic approach to deal with this is to use multivariate regression to correct for known biological and technical variables.

## 10 The future of gene expression analysis in Parkinson's

The technologies driving genome wide analysis are advancing rapidly [64,74]. As can be seen from this survey of the existing literature, the level of sophistication of gene expression studies in the PD brain has increased significantly even over the last 10 years. However, there are a number of newer technologies that have yet to be applied to this problem.

To date, published analyses of PD brains have used gene-by-gene expression arrays but there are newer techniques that apply array technology to events such as alternative splicing [75,88]. The importance of taking splicing into consideration in the context of neurological disease is highlighted by the presence of splicing mutations in the *MAPT* gene, leading to dementia and parkinsonism [56]. Variation at the *MAPT* locus has been identified as a risk factor for sporadic PD [104]. Measuring splicing with arrays has been used both for analysing brain tissue and in a disease setting [20,130]. One study using exon arrays has been carried out on blood isolated from PD patients and controls [103].

A very recently developed technology is deep sequencing, which can be applied to RNA quantification, called RNAseq [122]. The advantages of RNAseq over microarray-based measurements are that it is theoretically feasible to measure both expression levels and modifications such as splicing and RNA editing. There is some evidence that RNAseq data has a better dynamic range than arrays, and therefore is more predictive of true levels of gene expression, especially for lower abundance transcripts [125]. In principle, because RNAseq is a library based approach rather than based on designed oligonucleotide probes, there is also the possibility of discovery of novel transcripts. In the next several years, it is likely that there will be increasing use of RNAseq on samples from PD patients, as the cost of this technology decreases and it becomes more widely available. The issues regarding samples from diseased brains raised above will, however, apply equally to RNA sequencing as to microarray analysis, and so the challenge of working with PD brain samples will be just as large as for the lower resolution techniques. For high cost techniques, there is a tendency to pool samples or to use sample series of limited power, which is likely to be the major confound of early studies.

An alternative approach, which was touched upon by Noureddine and co-workers, combines data from genome wide association studies with gene expression data [85]. As the data from the current batch of large scale PD genome wide association studies becomes available, this is an approach that may yield a huge amount of useful information, as it does not necessarily rely upon the use of diseased tissue, instead examining gene expression in normal tissue of the genotype under investigation [23,40]. A cautionary note on this subject is that the size of effect of individual loci on lifetime risk for PD are modest at best, and it is likely that any alterations in gene expression that ensue from these genetic variations may also be modest. However, we have shown previously that there is evidence of similar expression quantitative trait loci for some, but not all, small effect loci.

A technique that offers another angle of approach to the issue of gene expression in PD is live cell analysis. Advances in the field of stem cell biology have opened the possibility of using neuronal cells derived from embryonic stem cells to model degeneration in an *ex vivo* environment [47]. This approach removes a number of the technical issues that plague analysis of mRNA from brain, while introducing a number of unknown quantities – for example it is not yet apparent how closely neuronal populations generated *ex vivo* match neuronal cells in an *in vivo* setting. With specific regard to PD, the application of induced pluripotent stem cell technology to sporadic and inherited forms of PD yields the potential to generate patient specific neuronal populations, which can then be used to examine gene expression [25,108,115]. Much remains to be discovered about how well human embryonic and induced pluripotent cells model the human condition, but this is a technique that may prove useful for future analyses of gene expression in PD.

**Fig. 1:**
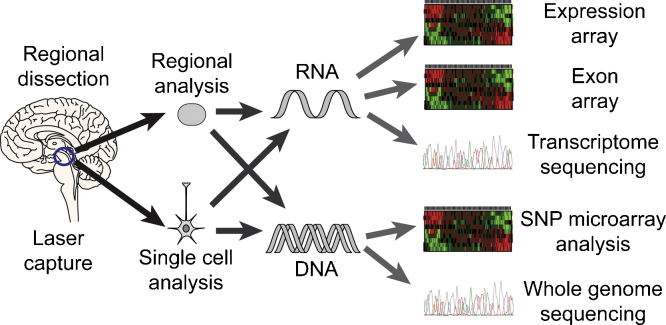
From brain tissue to genomic analysis. The flow of information from brain samples through the purification process and the different systems that can be used to analyse gene expression.

Finally, there are increasing opportunities to combine mRNA analyses with genome wide DNA sequence data and whole genome epigenetic mapping [34,54]. Again, this is perhaps best performed by examining gene expression at risk loci for a disease in control brains. We have shown that there are several risk loci for PD that are associated with differences in CpG methylation, particularly at the HLA locus on chromosome 6 [84]. The potential alternatives with regard to genomic analysis of a given sample of Parkinson's brain tissue are summarised in Fig. 1.

## 11 Good practices for gene expression studies in PD brain

Like all experiments, array studies require consideration of how to design and perform the experiments to yield interpretable data. The key recommendation, which has been made for other types of microarray studies, is that the power of a series to detect changes of a given magnitude should be considered when designing experiments. Given the need to correct for multiple testing, sample series might need to be relatively large to detect small differences and meta-analyses may be helpful. For this, deposition of raw data in public resources is encouraged.

Experimental variables should be controlled where possible, although for human *post mortem* studies there are practical constraints on ideal design. However, recognition that, statistically, some parameters are more important than other may help prioritize which aspects are more important. In an ideal design, samples from PD and controls would be ascertained from the same centre and would be hybridised in as few batches as possible, randomising cases and controls. Other known co-variates such as *post mortem* interval age and gender can be either matched and/or corrected for in the gene expression model used, but may have less influence on outcome of the experiment. However, RNA quality should be assessed, either by measuring brain pH or RIN, and it is helpful to only use the highest quality samples available.

Whether examining the nigra or other brain regions is most appropriate really depends on the question being asked. If the aim of the experiment is to examine the most heavily affected cells in PD then nigra is likely the best place to look and, in this context, LCM or other methods for isolating single cells is an extremely helpful technique. If the aim is to look more globally at the brain, then it is helpful to consider the proposed spread of pathology and have clinical data to hand that might reflect the involvement of different brain regions.

## 12 Conclusions

The application of techniques utilising a genomic approach to human biology and disease offer much promise in terms of opening a window on the processes underlying normal cellular and tissue function, and how these processes go awry in disease. Genome wide association studies have now demonstrated the utility of these approaches for PD, and a logical next step for these studies is their extension into the realm of gene expression. As gene expression studies of the brain in PD increase in size and complexity, it is likely that they will start to show the return that is already apparent for the association studies. When this is coupled to the increasing sensitivity and decreasing cost of genomic, transcriptomic and proteomic technologies, it is likely that the next few years will be an exciting time for the analysis of gene expression in PD.

## Conflict of interests

The authors have no conflicts of interest to declare.

## Figures and Tables

**Table 1:** Summary of genome wide gene expression analyses of Parkinson's disease brain tissue and cells.

Study	Year	Samples	Regions	Platform	Genes/pathways highlighted	Notes
Hauser et al.	2003	2 Control	SN	EST analysis		Used serial analysis of gene expression
Grunblatt et al.	2004	7 PD 7 Control	SN	Affymetrix HG focus, oligo array	Protein ubiquitination, SKP1A	
Lu et al.	2005	5 PD	SN DA neurons	RAP PCR fingerprinting	Cell survival genes	Compared Lewy body +ve and −ve neurons
Nourredine et al.	2005	3 PD 2 Control	SN	Serial analysis of gene expression	Following up on GWAS peaks from previous study	
Hauser et al.	2005	6 PD 2 PSP 1 FTDP 5 Control	SN	Affymetrix, HG U133A	Examined overlaps between disorders	Removal of overlapping genes allowed hierarchical segregation of gene expression phenotypes between different neurodegenerative disorders
Zhang et al.	2005	15 PD 15 Control	SN, putamen, Brodmann area 9	Affymetrix, HG U133A	Ubuiquitin proteasome system, electron transport chain	
Papapetropolis et al.	2006	22 PD 23 Control	21 brain regions	Affymetrix, HG U133A	MRPS6 (Mitochondrial ribosomal protein S6)	
Lu et al.	2006	5 Control	DA neurons	EST analysis	Cell survival pathways	LCM – compared midbrain DA neurons to central grey substance DA neurons
Vogt et al.	2006	4 PD 4 MS 4 Control	Putamen, cerebellum, occipital cortex	Affymetrix, HG U133A	G protein signalling and transcriptionalregulation	1 MSA specific transcript dysregulated: FAM49A
Miller et al.	2006	6 PD 8 Control	SN, striatum	GE Codelink 20k bioassay	Synaptic genes	Principal component analysis shows segregation between PD and control
Moran et al.	2006	15 PD 8 Control	SN	Affymetrix, HG U133A and B	DNAJB1	Same dataset as Moran et al. [134] and Duke et al. [135]
Moran et al.	2007	13 PD 2 PDD 8 Controls	SN	Affymetrix, HG U133	a synuclein, dopamine and parkin pathways	1 case PDD, 1 case had concomitant pathological diagnosis of AD
Grunblatt et al.	2007	13 AD 9 PD 9 Controls	Hippocampus, cerebellum, Gyrus frontalis medialis	Affymetrix, HG U133A and B	Cannabinoid receptor 2, Histone, cluster 1 H3e, nicotinic cholinergic, receptor a6, bAPP cleaving enzyme 1	
Cantuti-Castelvetri et al.	2007	8 PD 8 Control	SN, DA neurons	Affymetrix, HG X3P	Gender specific transcriptional profiles, proteolysis, WNT pathway, a synuclein, PINK1	LCM, 500 DA neurons from each brain.
Duke et al.	2007	9 PD 7 Control	SN	Affymetrix, HG U133A and B	Glial specific genes	Compared lateral and medial SN to identify genes linked to survival of medial SN
Langerveld et al.	2007	7 MSA 5 Controls	Rostrol pons	Affymetrix, HG U133A	Mitochondrial function, ubiquitin, proteasome, inflammation	
Stamper et al.	2008	15 PD 13 PDD 14 Control	Posterior cingulate, cortex pytamidal, neurons	Affymetrix HG U133 + 2.0	mRNA splicing genes	LCM of 1000 neurons per brain
Bossers et al.	2009	4 PD 4 PDD 8 Control	SN, caudate, nucleus, putamen	Custom Agilent 22k arrays	Neurotrophic support and axon guidance	Hierarchical segregation of PD vs control in SN but not other tissues
Simunovic et al.	2009	10 PD 9 Control	SN DA neurons	Affymetrix HG U133A	Mitochondria, ubuiquitin proteasome system and programmed cell death	LCM, 300 neurons from controls, 700 from PD samples
Elstners et al.	2009	8 PD 9 Control	SN, DA neurons	Illumina WG6v1	MTND2, PDXK, SRGAP3, TRAPPC4. PDXK, pyroxidal kinase, linked to increased risk of PD	LCM, 100 neurons per brain
Zheng et al.	2010	172 PD 139 Control	SN, DA neurons	Meta analysis	Electron transport chain genes under the control of PGC1a	Included LCM data and a series of subclinical samples
Simunovic et al.	2010	10 PD 9 Control	SN, DA neurons	Affymetrix, HG U133A	Gender specific transcriptional profiles	Used same data set as Simunovic et al. [105]
